# Comparison of diagnostic methods to detect *Histoplasma capsulatum* in serum and blood samples from AIDS patients

**DOI:** 10.1371/journal.pone.0190408

**Published:** 2018-01-17

**Authors:** Katia Cristina Dantas, Roseli Santos de Freitas, Marcos Vinicius da Silva, Paulo Ricardo Criado, Olinda do Carmo Luiz, Adriana Pardini Vicentini

**Affiliations:** 1 Department of Pathology, Sao Paulo University Medical School, Sao Paulo, Brazil; 2 Medical Mycology Laboratory-LIM 53/HCFMUSP and Institute of Tropical Medicine, University of Sao Paulo, Sao Paulo, Brazil; 3 Emilio Ribas Institute of Infectious Diseases, Consultant, Ministry of Health, Department of Medicine, Catholic University of Sao Paulo, and Professor, Program in Postgraduate Sciences and Coordination of Disease Control, Department of State Health, Sao Paulo, Brazil; 4 Full Researcher at ABC Medical School, Sao Paulo, Brazil; 5 Preventive Medicine Department, Sao Paulo University Medical School, Sao Paulo, Brazil; 6 Immunology Center, Adolfo Lutz Institute, São Paulo, Brazil; Waseda University, JAPAN

## Abstract

**Background:**

Although early and rapid detection of histoplasmosis is essential to prevent morbidity and mortality, few diagnostic tools are available in resource-limited areas, especially where it is endemic and HIV/AIDS is also epidemic. Thus, we compared conventional and molecular methods to detect *Histoplasma capsulatum* in sera and blood from HIV/AIDS patients.

**Methodology:**

We collected a total of 40 samples from control volunteers and patients suspected of histoplasmosis, some of whom were also infected with other pathogens. Samples were then analyzed by mycological, serological, and molecular methods, and stratified as histoplasmostic with (group I) or without AIDS (group II), uninfected (group III), and infected with HIV and other pathogens only (group IV). All patients were receiving treatment for histoplasmosis and other infections at the time of sample collection.

**Results:**

Comparison of conventional methods with nested PCR using primers against *H*. *capsulatum* 18S rRNA (HC18S), 5.8S rRNA ITS (HC5.8S-ITS), and a 100 kDa protein (HC100) revealed that sensitivity against sera was highest for PCR with HC5.8S-ITS, followed by immunoblotting, double immunodiffusion, PCR with HC18S, and PCR with HC100. Specificity was equally high for double immunodiffusion, immunoblotting and PCR with HC100, followed for PCR with HC18S and HC5.8-ITS. Against blood, sensitivity was highest for PCR with HC5.8S-ITS, followed by PCR with HC18S, Giemsa staining, and PCR with HC100. Specificity was highest for Giemsa staining and PCR with HC100, followed by PCR with HC18S and HC5.8S-ITS. PCR was less efficient in patients with immunodeficiency due to HIV/AIDS and/or related diseases.

**Conclusion:**

Molecular techniques may detect histoplasmosis even in cases with negative serology and mycology, potentially enabling early diagnosis.

## Introduction

*Ajellomyces capsulatus* (anamorph *Histoplasma capsulatum*), a dimorphic fungus that takes a saprophytic mycelial form in the soil but a pathogenic yeast form in the host lung [1,2], causes histoplasmosis, a widely distributed systemic mycotic infection. However, histoplasmosis is significantly more prevalent in immunocompromised individuals, especially among HIV or AIDS patients who have limited access to antiretroviral therapy. In addition, the mortality rate among HIV/AIDS patients diagnosed with histoplasmosis is 30% in Latin America, but only 4–8% in the United States [3,4]. Histoplasmosis is particularly common in Brazil, where it is the second most frequent invasive fungal infection in HIV/AIDS patients and results in high mortality [5,6]. According to Ostrosky-Zeichner [7], early diagnosis of invasive fungal infections is critical, as delays often render antifungal therapy ineffective or even cause death.

Histoplasmosis is traditionally and directly diagnosed by histopathology using specific stains, as well as by isolation of the fungus in culture, which is considered the gold standard [1]. Indirect immunological assays to detect antibodies and/or antigens are also valuable [1,8]. In any case, both direct and indirect assays vary in sensitivity and specificity depending upon the method, clinical form of the disease, and immune status of the host [8,9,10]. More recently, molecular techniques have gained prominence due to greater speed, sensitivity, and specificity [1,8,11]. Although perhaps not yet routinely employed, these methods include double immunodiffusion, counterimmunoelectrophoresis [12], and PCR [13,14]. Indeed, PCR methods were recently developed based on blood samples spiked with *H*. *capsulatum* DNA [15], as well as on sera and whole blood from histoplasmosis patients [16]. The aim of this study was to compare conventional, *i*.*e*., mycology and serology, and molecular methods to detect *H*. *capsulatum* in sera and blood from patients with AIDS, with a view to assist clinicians in early diagnosis and choice of therapy.

## Methods

### Ethics statement

The study was approved by institutional ethics committees at University of São Paulo Medical School Hospital (no. 0372/09), Institute Adolfo Lutz (no. 007/2010), and Emilio Ribas Institute of Infectious Diseases (no. 348/2009).

### Patients

Blood samples (n = 40) were collected between January 2009 and December 2011 from patients admitted with suspected histoplasmosis to the emergency units at Emilio Ribas Institute of Infectious Diseases and Clinical Medical Hospital at University of Sao Paulo Medical School. The samples were tested at both institutions for HIV, hepatitis, syphilis, *Paracoccidioides brasiliensis*, *Cryptococcus neoformans*, *Aspergillus fumigatus*, and *Histoplasma* sp. In addition, samples were analyzed by culture and direct microscopy on Sabouraud glucose agar. After results were obtained from mycological (positive or negative) and serological (reactive or nonreactive) assays, patients were requested to participate in this study, and those who agreed were asked to fill out a relevant questionnaire and sign a form indicating informed consent. Patients who were pregnant or younger than 18 years were excluded. After diagnosis, samples were classified as histoplasmotic with AIDS (group I, n = 12) or without AIDS (group II, n = 8), uninfected (group III, n = 10), or infected with HIV and other pathogens only (group IV), including *P*. *brasiliensis* (n = 2), *C*. *neoformans* (n = 2), *Aspergillus* spp. (n = 2), *Leishmania* (n = 2), and rheumatoid factor (n = 2). In cases where *Histoplasma* was not isolated from patient samples, diagnosis was confirmed by histopathology or, in some cases, by autopsy.

### Control strains

To establish diagnostic sensitivity and specificity, heterologous control strains were selected based on clinical similarity to *H*. *capsulatum*, and consisted of *P*. *brasiliensis* 18 and B-339 (ATCC 32069), *Candida albicans*, *C*. *parapsilosis*, *C*. *neoformans* ATCC 24067, and *Aspergillus* spp., all of which were obtained from Micoteca do Instituto de Medicina Tropical de Sao Paulo. Positive control strains consisted of *H*. *capsulatum* ATCC 28308 (CDC: B973), ATCC 12700 (CDC: A811), and HC200 (GenBank: DQ239887).

### Mycology

Giemsa-stained smears were observed by direct microscopy for oval elements in phagocytes that are 3–4 μm in diameter with typical cap coloration (nuclear chromatin at poles) and small, surrounding light halos (false capsules). Smears were prepared from serial blood samples collected and maintained under sterile conditions and inoculated on Sabouraud-Dextrose agar (Difco Laboratories, Detroit, MI), Brain-Heart Infusion agar (Difco Laboratories, Detroit, MI), and tryptone soya broth (Oxoid, London, England). Cultures were incubated at 35°C, and pathogen growth was assessed for 60 days.

### Serology

Double immunodiffusion and immunoblotting were performed according to Freitas et al. [17] and Passos et al. [18], respectively, with some modifications.

### DNA extraction

To extract DNA from cell cultures, 200 μL samples were mixed with 40 μL of 60 mg/mL lysing enzymes from *Trichoderma harzianum* (cat. no. L1412, Sigma Chemical Co., St. Louis, MO, USA) in 1 M sorbitol, 100 mM EDTA, and 14 mM β-mercaptoethanol. Samples were then incubated for 30 min at 30°C and centrifuged at 5,000 ×*g* (Eppendorf, Hamburg, Germany) at room temperature. Precipitated cells were resuspended in 180 μL of ATL buffer (QIAamp DNA Mini Kit, Qiagen, Hilden, Germany), and lysed for 3 h at 56°C with 100 mg/mL proteinase K. DNA was then extracted using QIAamp DNA Mini Kit. To extract DNA from serum and blood, 200 μL samples were lysed for 3 h at 56°C with 100 mg/mL proteinase K, and DNA was then extracted using QIAamp Blood DNA Mini Kit (Qiagen).

### Nested PCR

The presence of amplifiable DNA was confirmed by nested PCR of a fragment of human glyceraldehyde-3-phosphate dehydrogenase (*GADPH*; GenBank: J04038.1), as described previously [19]. Outer primers 5' GAC AAC AGC CTC AAG ATC ATC 3' and 5' GAC GGC AGG TCA GGT CCA CCA 3' were used to amplify a 610 bp fragment, and inner primers 5' AAT GCC TCC TGC ACC ACC 3' and 5' ATG CCA GTG AGC TTC CCG 3' were then used to amplify an internal 248 bp product. In the first round, targets were amplified from 2 μL DNA extract in 25 μL of 10 mM Tris-HCl pH 8.3, 50 mM KCl, 2.5 mM MgCl_2_, 0.3 μM each of outer primers, 1.5 U AmpliTaq DNA polymerase, and 100 μM of each dNTP, over one cycle at 94°C for 5 min, 35 cycles at 94°C for 30 s, 56°C for 30 s, and 72°C for 45 s, and final extension at 72°C for 5 min. In the second round, targets were amplified from 2 μL of the initial amplification product in 50 μM of each dNTP and 0.3 μM of each inner primer, over the same thermal profile as the first reaction, except that 40 cycles were carried out. Positive and negative controls without DNA were included in all assays.

*H*. *capsulatum* 18S rRNA gene (HC18S) was amplified according to reaction conditions adapted from Bialek et al. [20]. Briefly, outer primers 5' GTT AAA AAG CTC GTA GTT G 3' and 5' TCC CTA GTC GGC ATA GTT TA 3' were used to amplify a 429 bp sequence from several fungi that are pathogenic to humans. Inner primers 5' GCC GGA CCT TTC CTC CTG GGG AGC 3' and 5' CAA GAA TTT CAC CTC TGA CAG CCG A 3' were then used to amplify a 231 bp sequence specific to *Histoplasma* spp. Reaction conditions for a 100 kDa *H*. *capsulatum* protein (HC100) were similarly adapted from Bialek et al. [21]. In particular, outer primers 5' GCG TTC CGA GCC TTC CAC CTC AAC 3' and 5' ATG TCC CAT CGG GCG CCG TGT AGT 3' were used to amplify a 391 bp sequence, and inner primers 5' GAG ATC TAG TCG CGG CCA GGT TCA 3' and 5' AGG AGA GAA CTG TAT CGG TGG CTT G3' were then used to amplify a 210 bp sequence specific to *Histoplasma* were amplified in 25 μL reactions as described previously [20,22]. In the first round, reactions consisted of 2 μL DNA extract, 10 mM Tris-HCl pH 8.3, 50 mM KCl, 2.5 mM MgCl_2_, 1 μM of each outer primer, 1.5 U of Platinum Taq DNA poly Brazil, and 100 μM of each dNTP. The thermal profile consisted of one cycle at 94°C for 5 min, 35 cycles at 94°C for 30 s, 50°C (HC18S) or 65°C (HC100) for 30 s, and 70°C for 1 min, and one cycle at 72°C for 5 min. Reaction mixtures for the second round were identical, except that 1 μL of the first reaction product, 50 μM dNTP, and 1 μM of each outer primer were used. The thermal profile in this round consisted of one cycle at 94°C for 5 min, 30 cycles at 94°C for 30 s and 72°C for 1 min, and then one cycle at 72°C for 5 min. High annealing temperatures were used in this round to enhance stringency.

For nested PCR of *H*. *capsulatum* 5.8S rDNA ITS (HC5.8S-ITS), all strains were first sequenced with primers 5' TCC GTA GGT GGA CCT GCG 3', 5' GCA TCG ATG AAG AAC GCA GC 3', and 5' TCC TCC GCT TAT TGA TAT GC 3', to target the conserved 18S, 5.8S, and 28S regions of the rRNA gene [22]. ITS1 and ITS4 were then used to amplify the intervening HC5.8S-ITS sequence in 25 μL as described previously [22], using conditions described in Fujita et al. [23]. In the primary round, reactions consisted of 2 μL DNA extract in 10 mM Tris-HCl pH 8.3, 50 mM KCl, 2.5 mM MgCl_2_, 1 μM outer primers, 1.5 U Platinum Taq DNA poly Brazil, and 100 μM each of dNTP. Targets were amplified over one cycle at 94°C for 5 min, 35 cycles at 94°C for 30 s, 65°C for 30 s, and 72°C for 1 min, and one cycle at 72°C for 5 min. Reaction mixtures in the second round were identical, except that 2 μL of the first reaction product, 50 μM dNTP, and 1 μM each of inner primers were used.

All PCR reagents were obtained from Invitrogen (Carlsbad, CA, USA), and samples were processed and amplified three times on a Veriti 96 thermocycler (Applied Biosystems, Life Technologies Corporation, Carlsbad, CA, USA). To avoid contamination of components, preparation of reaction mixtures and addition of template DNA were performed in separate rooms. All assays included negative controls without DNA and positive controls with DNA from *H*. *capsulatum* ATCC A811 and B923, *C*. *neoformans* ATCC 24067, and *P*. *brasiliensis* 18 and 339. Products were electrophoresed on 1.5% agarose, stained with ethidium bromide, and visualized on a UV transilluminator.

PCR products were purified with PureLink Kit (Invitrogen), and sequenced according to manufacturer protocols on a MegaBACE 1000, a system with 96 capillaries, using DYEnamic ET Dye Terminator Kit with Thermo Sequence II DNA polymerase (GE Healthcare formerly Amersham Biosciences, Marlborough, MA, USA). Pathogens were identified by BLAST (http://www.ncbi.nlm.nih.gov/Blast) against GenBank.

### Combined tests

Direct observation was used to identify tests that would provide greater sensitivity when performed in parallel. A combined test was considered positive when either of the tests performed in parallel were positive. Fungal isolation was considered the gold standard for diagnosis failed, histopathology was used to confirm histoplasmosis.

### Statistical analysis

Data obtained from conventional (mycology and serology) and molecular (nested PCR) assays were analyzed according to Fletcher et al. [24] to determine sensitivity, specificity, positive predictive value, negative predictive value, and accuracy. Results from *H*. *capsulatum* cultures, histopathology or, in some cases, by autopsy were used as reference. Agreement between the reference method and other methods was assessed by inter-rater agreement (Cohen's Kappa) [25], as interpreted using Landis and Koch-Kappa Benchmark Scale [26]. Marginal homogeneity was assessed using McNemar's test.

## Results

The study population consisted of biological samples from 20 individuals suspected to have histoplasmosis and 10 uninfected individuals. Patients with disseminated histoplasmosis (group I) presented high fever, diarrhea, weight loss, generalized lymphadenopathy, hepatosplenomegaly, neurological symptoms, acute renal failure, respiratory failure, and skin lesions, and 100% of these patients had CD4 lymphocytes fewer than 200 cells/mm^3^. Other infections detected in some group I patients are described in Table 1. Of the 8 patients in Group II, 2 presented disseminated diseases, 2 presented acute pulmonary histoplasmosis, 1 presented subacute pulmonary histoplasmosis, 2 presented chronic pulmonary histoplasmosis, and 1 presented supra renal histoplasmosis. These patients also presented respiratory tract infections, fever, headache, cough, night sweats, weight loss, chest pain, neurological symptoms, and abdominal pain. Patients with histoplasmosis were often mistaken as having tuberculosis, as well as having other associated diseases (Table 1).

**Table 1:** Clinical and pathological characteristics of patients in groups I and II.

Group	Patients	Age	Sex	Clinical Formal	Association Disease
GI - Histoplasmosis and AIDS	1	37	F	Disseminated Histoplasmosis	Tuberculosis / Neurotoxoplasmosis
	2	38	M	Disseminated Histoplasmosis	Meningitis–Candidiasis
	3	40	M	Disseminated Histoplasmosis	Tuberculosis / Leishmaniosis / Hepatitis C
	4	46	M	Disseminated Histoplasmosis	Tuberculosis / Hepatitis C
	5	56	F	Disseminated Histoplasmosis	Pneumocistosis
	6	35	M	Disseminated Histoplasmosis	Candidiasis
	7	25	F	Disseminated Histoplasmosis	Tuberculosis
	8	41	M	Disseminated Histoplasmosis	Tuberculosis
	9	49	M	Disseminated Histoplasmosis	Disseminated Infections
	10	46	M	Disseminated Histoplasmosis	Tuberculosis
	11	66	M	Disseminated Histoplasmosis	Disseminated Infections
	12	40	F	Disseminated Histoplasmosis	Meningitis
GII Histoplasmosis	1	32	M	Acute pulmonary histoplasmosis	Cancer
	2	27	F	Disseminated Histoplasmosis	Sepsis
	3	59	F	Chronic Pulmonary Histoplasmosis	Pneumonia—Tuberculosis
	4	33	M	Acute Pulmonary Histoplasmosis	Diabetes Mellitus
	5	45	M	Chronic Pulmonary Histoplasmosis	Tuberculosis
	6	41	M	Subacute pulmonary histoplasmosis	Meningitis
	7	42	M	Supra renal histoplasmosis	Renal Insufficiency
	8	43	M	Disseminated Histoplasmosis	Sepsis

AIDS—Acquired Immunodeficiency Syndrome; F—Female; M–Male

**Fig 1:**
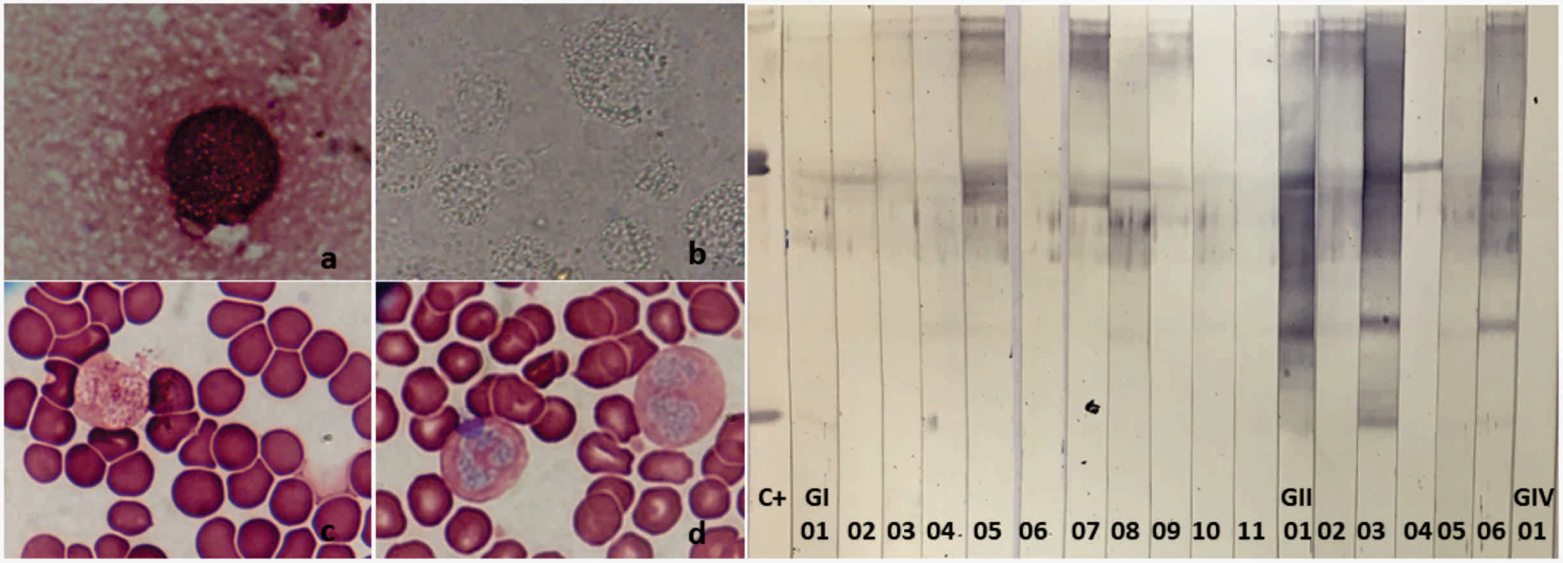
Mycological and serological tests for histoplasmosis. **A**, Mycological tests suggestive of histoplasmosis in a sample from a histoplasmotic patient with HIV. a, Pleural fluid stained by Giemsa, 1,500×; b, intracellular yeast in alveolar macrophages with cytoplasmic retraction by direct exams, 1,500×; c and d, blood smear stained with Giemsa, showing basophil nuclei and intracellular yeasts with cytoplasmic retraction. **B**, Immunoblotting for circulating *H*. *capsulatum* antibodies in sera from histoplasmotic patients with (GI) and without HIV (GII), and from patients with HIV or other infections only (GIV). C+, polyclonal *H*. *capsulatum* antibody (positive control); 1–10, sera from patients with suspected histoplasmosis. Fractions H (108–120 kDa) and M (70 and 94 kDa) are indicated.

Histoplasmosis was confirmed by mycology in 83.33% and 12.5% of patients with and without HIV, respectively (Table 2, Fig 1), with *H*. *capsulatum* isolated from 50% and 25% of blood samples. However, one isolate suggestive of histoplasmosis based on Giemsa staining was subsequently identified as *Candida glabrata*. Control strains were also characterized by conventional and molecular methods, and were found by sequencing of HC5.8S-ITS to be at least 98% identical to reference species.

**Table 2:** Comparison of methods to detect *H*. *capsulatum* among groups.

Test	Method	Sensitivity (%)	Specificity (%)	Accuracy (%)	PPV (%)	NPV (%)	Kappa	MacNemar’s test Exact P
Mycological	Giemsa staining	55	100	77.5	100	69	0.159	0.004
	Fungal isolation	-	-	-	-	-	0.111	0.001
Serological	DI	25	100	-	100	52	0.053	0.000
	IB	50	100	52.5	100	66.66	0.143	0.002
Molecular	HC18S blood	60	90	75	86	70	0.697	0.109
	HC18S serum	25	85	75	62.5	53	0,500	0.007
	HC100 blood	54	100	55	100	60.6	0,644	0.000
	HC100 serum	18	100	68	100	54	0,558	0.000
	HC5.8-ITS blood	70	80	58	78	73	0,677	0.754
	HC5.8-ITS serum	65	80	75	76	70	0,651	0.549
Combined test	HC18S blood HC5.8-ITS serum Fungal isolation	90	90	72.5	90	90	0.754	1.000

DI—Double Immunodiffusion; IB—immunoblotting; HC18 blood primers - 18S rRNA of *H*. *capsulatum*; HC 100 primers—100 kDa of *H*. *capsulatum* protein; HC5.8-ITS primers—5.8S rDNA ITS *of H*. *capsulatum*; %—percentage; PPV—positive predictive value; NPV -, negative predictive value.

Serology by double immunodiffusion revealed that 25% and 12.5% of histoplasmotic patients with and without HIV, respectively, had circulating *H*. *capsulatum* antibodies. All other patients did not react against *H*. *capsulatum* antigens. On the other hand, 66.7% and 25.0% of samples from histoplasmotic patients with or without HIV reacted with *H*. *capsulatum* H and M fractions on immunoblots, while samples from all other patients did not (Table 2, Fig 1).

Nested PCR against the housekeeping gene *GADPH* was used to test for the presence or absence of amplifiable DNA, as well as for the presence of PCR inhibitors (Table 2). Against sera from histoplasmotic patients with HIV, the sensitivity of nested PCR was highest using HC5.8S-ITS primers (92%), followed by HC18S primers (34%), and HC100 primers (25%). However, but was still highest for HC5.8S-ITS primers (25%), followed by HC18S (12.5%) primers. *H*. *capsulatum* DNA was undetectable in these patients using HC100 primers. Against blood from histoplasmotic patients with HIV, sensitivity was also highest for HC5.8S-ITS primers (91.66%), followed by HC18S primers (66.6%), and HC100 primers (33.3%). The sensitivity profile was again different in histoplasmotic patients without HIV, and was highest for HC18S primers (50%) but comparable (37.5%) for HC100 and HC5.8S-ITS primers (Table 2).

All three-primer pairs exhibited 100% specificity when tested against uninfected sera and blood samples. Among patients without histoplasmosis but with HIV and other infections, 100% specificity was achieved only with HC100 primers. On the other hand, specificity was 60% in sera and blood samples tested with HC5.8S-ITS primers, and 70% in sera and 80% in blood samples tested with HC18S primers.

All samples from histoplasmotic patients with or without HIV were analyzed by sequencing, and results confirmed the presence of *H*. *capsulatum* with 98% identity to reference strains. A strain of *C*. *glabrata* was isolated in culture from a histoplasmotic patient without HIV (sample 1), along with a strain of *H*. *capsulatum* from another patient in the same group (sample 4). Sequences from the latter strain were 97% identical to those of *Pichia kudriavzevii* and 99% identical to those of *H*. *capsulatum*. No fungal cells were isolated from samples 7 and 8 of histoplasmotic patients without HIV; however, sequences derived from these blood samples were 98% identical to *H*. *capsulatum* and 99% identical to *Rhodotorula mucilaginosa*, respectively. Sequencing was not possible for strains 2, 3, 5, and 6.

Among all methods, sensitivity for *H*. *capsulatum* in sera was highest for PCR with HC5.8S-ITS, followed by immunoblotting, by double immunodiffusion and PCR with HC18S, which have equal sensitivity, and, finally, by PCR with HC100. Against blood, sensitivity was also highest for PCR with HC5.8S-ITS, followed by PCR with HC18S, Giemsa staining, and PCR with HC100. In contrast, specificity was equally high against sera for double immunodiffusion, immunoblotting, and PCR with HC100, followed by PCR with HC18S and HC5.8S-ITS. On the other hand, specificity against blood was highest for Giemsa staining and PCR with HC100, followed by PCR with HC18S and HC5.8S-ITS (Table 2). Positive predictive value against sera was equally high for double immunodiffusion, immunoblotting, and PCR with HC100, followed by PCR with HC5.8S-ITS and then by PCR with HC18S. Negative predictive value was highest for PCR with HC5.8S-ITS, followed by immunoblotting, PCR with HC100, PCR with HC18S, and double immunodiffusion (Table 2). Against blood, positive predictive value was highest for both Giemsa staining and PCR with HC100, followed by PCR with HC18S, and then by PCR with HC5.8S-ITS, while negative predictive value was highest for PCR with HC5.8S-ITS, followed by PCR with HC18S, Giemsa staining, and PCR with HC100 (Table 2).

Direct observation of data was used to verify that sensitivity was greater for the following combination of tests: fungal isolation and PCR with HC5.8S-ITS, and fungal isolation and PCR with HC18S against blood. For these combined tests, the sensitivity, specificity, and negative and positive predictive values were 90%.

Kappa analysis confirmed substantial agreement of the results of HC18 against blood, HC5.8 against blood, HC100 against blood, and HC5.8 against serum with the results of the gold standard, while HC18 and HC100 against serum showed moderate agreement and the other tests showed slight agreement (Table 2).

Analysis using McNemar’s test indicated that results of HC5.8-ITS against blood and serum, HC18S against blood, and the combined test did not differ significantly from those of the gold standard.

## Discussion

Bahr et al. [27] argued that, as a consequence of HIV pandemicity, progressive disseminated histoplasmosis has grown more prevalent not only in known endemic regions, but also in areas not considered endemic. The increasingly expanding suite of immunosuppressive medications and biologics has also compounded this trend, which appears to be independent of geographic location or patient travel. However, histoplasmosis remains challenging to diagnose, as the turnaround time for a positive culture, the current gold standard of diagnosis, can be significant [1]. Hence, we compared various diagnostic methods against blood and sera collected from infected patients.

Direct microscopy and other mycological assays may only be suggestive but not conclusive of histoplasmosis, owing to the similarity in structure between *H*. *capsulatum* yeast and other pathogens, which can lead to false positives [1,5]. Indeed, *H*. *capsulatum* is difficult to differentiate by histopathology and microscopy from other yeasts such as *C*. *glabrata* and other *Candida* species, as well as from diminutive forms of other pathogens such as *Cryptococcus*, *P*. *brasiliensis*, *Pneumocystis jirovecii*, and even from protozoa such as *Leishmania donovani* and *Toxoplasma gondii* [5]. In addition, Guimarães et al. [5] reported that the sensitivity of microscopy (Giemsa staining) and histopathology in histoplasmostic patients with limited, acute/subacute, chronic, disseminated pulmonary, or mediastinal HIV/AIDS are 9%, 10%, 17–40%, 43%, and < 25%, respectively, indicating low sensitivity in HIV/AIDS and possibly in other immunocompromised patients.

*H*. *capsulatum* isolated *in vitro* may also exhibit similar morphology as non-pathogenic species like *Chrysosporium*, *Corynascus*, *Renispora*, and *Sepedonium*. There are also atypical *H*. *capsulatum* isolates that may prevent accurate identification [28]. In this study, fungi suggestive of *H*. *capsulatum* were isolated from 50% and 25% of histoplasmotic patients with and without HIV, respectively, and confirmed by morphology in 27% and 12.5%, respectively. Of note, sample 3 among patients with HIV revealed co-infection with *C*. *albicans*, which was found to be predominant based on sequencing. Collectively, the data confirm that cultures have low sensitivity in histoplasmotic patients with or without HIV [29]. Moreover, *in vitro* isolation of *H*. *capsulatum* from patients with HIV/AIDS may be inhibited by administration of sulfamethazole-trimethoprim to treat lung infections, primarily those caused by *P*. *jirovecii* [30]. Indeed, we found that all isolates were inhibited by sulfamethazole-trimethoprim. Nevertheless, this drug is effective against paracoccidioidomycosis, and is often the treatment of choice depending on socioeconomic conditions. We note, however, that the drug is not used in Brazil to treat histoplasmosis.

Of existing serological assays, double immunodiffusion is most often used in the clinic. This technique is inexpensive, but has variable sensitivity and specificity, with predictive values 86–100% depending on the antigen used. It also enables evaluation of therapeutic effectiveness based on titers of specific fungal antibodies [12,31]. However, double immunodiffusion has low sensitivity in immunocompromised patients who produce immunoglobulins at reduced levels [32,33]. We found that 25% of sera from histoplasmotic patients with HIV tested positive for *H*. *capsulatum* antibodies on double immunodiffusion, with titers ranging from 1:4 to 1:16. However, 66.7% (8/12) of samples testing negative on double immunodiffusion subsequently tested positive on immunoblots. Of these samples, five reacted more strongly with the M fraction than with the H fraction, indicating active disease. On the other hand, results from both assays were consistent for four samples. Among histoplasmotic patients without HIV, *H*. *capsulatum* antibodies were detected on double immunodiffusion in 12.5% of sera, with titers 1:4, while 25% tested positive on immunoblots. Results were consistent between methods for 12.5% of these samples. Accordingly, sensitivity was 25% for double immunodiffusion and 50% for immunoblotting against histoplasmotic patients with or without HIV. These low percentages are due to the general inability of patients with AIDS and other severe diseases to mount an adequate antibody response to circulating antigens [32,33]. Moreover, the potential for false-positives and cross-reactivity with other pathogens such as cutaneous leishmaniasis is a serious limitation. Non-specific reactivity has been attributed to carbohydrate C, a thermostable galactomannan found in most systemic dimorphic fungi [34].

Blood was collected from histoplasmotic patients during hospitalization and antifungal therapy. Although the double immunodiffusion methodology presents a high degree of specificity, its sensitivity is moderate. It should also be noted that some of these patients had circulating *H*. *capsulatum* titers that were below the detection limit for the methodology. Therefore, we propose that immunoblotting and/or PCR be included in the methodology as confirmatory tests.

In contrast, several studies using specific PCR primers have demonstrated high sensitivity and specificity for histoplasmosis. Samples evaluated using these primers have included isolated fungal cultures [35], whole blood [16], and paraffin-embedded tissues [20,21,36]. However, DNA-based detection of *H*. *capsulatum* has not yet been validated as a diagnostic tool, and is not commercially available [8,10]. Results using HC100 primers demonstrated 100% specificity and reliability in total blood and serum, confirming previous results [15,16,37]. Indeed, Ohno et al. [38] demonstrated that these primers have great potential in initial diagnosis, with high sensitivity, 90% specificity against blood, and 85% specificity against sera, but emphasized the need for concurrent use of conventional methods. HC18S primers performed better against blood, and had higher specificity and positive predictive value than HC5.8S-ITS. The specificity of the former was similarly higher against sera, although the positive predictive value was higher for the latter. Nevertheless, both primers exhibited relatively lower specificity due to genomic similarity between *H*. *capsulatum* and other species such as *P*. *brasiliensis* and *Aspergillus fumigatus*. This explains false positives observed in patients who only have HIV, aspergillosis, cryptococcosis, or paracoccidioidomycosis.

The generally low sensitivity of all methods tested against blood and sera from patients with HIV/AIDS confirms previous findings by Buitrago et al. [39], Toranzo et al. [40], and Frías-De-León et al. [37]. In particular, PCR-based methods exhibited lower efficacy against patients with immunodeficiency, presumably because of ongoing treatments for histoplasmosis and/or other infections.

The association of histoplasmosis with other pathologies such as pulmonary or disseminated tuberculosis, neurological disorders, cancer, diabetes mellitus, hypertension, and infection with other fungi, viruses, or parasites (e.g., toxoplasmosis, leishmaniasis) significantly complicates the detection of the histoplasmosis. Nevertheless, we recommend the use of PCR with rDNA primers in conjunction with conventional methods, especially since PCR is faster than culture, and does not require handling of infectious fungi. In addition, these tools may enable early diagnosis, even in cases of negative serology and mycology. We note, however, these methods remain in-house, with limited availability and without independent validation.

Although further studies are needed, our results indicate that using a combination of tests may increase diagnostic capacity. Sensitivity may be increased by simultaneously performing HC18 against blood, HC5.8 against serum, and fungal isolation to identify histoplasmosis. For such combinations, the sensitivity, specificity, and negative and positive predictive value were 90%. However, to resolve the occurrence of 10% false negatives, we suggest further confirmatory analysis of negative results with a more specific combined test, such as HC100 primers (blood and serum), Giemsa staining, DI, and IB, which presented 100% specificity.
